# New histone deacetylase inhibitors improve cisplatin antitumor properties against thoracic cancer cells

**DOI:** 10.18632/oncotarget.2056

**Published:** 2014-06-03

**Authors:** Fabien Gueugnon, Pierre-François Cartron, Cedric Charrier, Philippe Bertrand, Jean-François Fonteneau, Marc Gregoire, Christophe Blanquart

**Affiliations:** ^1^ Inserm, U892, F-44000, Nantes, France; ^2^ CNRS, UMR6299, F-44000, Nantes, France; ^3^ Université Nantes, F-44000, Nantes, France; ^4^ Réseau Epigénétique du Canceropôle Grand Ouest; ^5^ CNRS, UMR7285, Institut de Chimie des Milieux et Matériaux de Poitiers, Université de Poitiers, France

**Keywords:** Mesothelioma, lung cancer, chemotherapy and epigenetic

## Abstract

Histone deacetylase inhibitors (HDACi) have shown promising antitumor effects on numerous cancer cells including malignant pleural mesothelioma (MPM) and lung adenocarcinoma (ADCA) cells. However, clinical trials using these compounds alone have shown limited efficacy against solid tumors. Therefore, new molecules are being developed and combinations with classical chemotherapeutic drugs are being tested.

Here, we have evaluated on three MPM and three lung ADCA cell lines the antitumor potential of four new HDACi compounds, either alone or in combination with cisplatin. These effects were compared with those of vorinostat, an HDACi approved for cancer treatments.

First, we characterized the HDAC mRNA expression profiles of tumor cells and showed an increase of the classI/classII HDAC ratio. We then treated cancer cells with these new HDACi and observed a cell-death induction and an increase of HDACi target genes and proteins expression. This was particularly evident for NODH compound (pan-HDACi) which had similar effects at nanomolar concentrations as micromolar concentrations of vorinostat. Interestingly, we observed that the HDACi/cisplatin combination strongly increased cell-death and limited resistance-phenotype emergence as compared with results obtained when the drugs were used alone.

These results could be exploited to develop MPM and lung ADCA treatments combining chemotherapeutic approaches.

## INTRODUCTION

Malignant pleural mesothelioma (MPM) is considered to be one of the worst cancers in terms of clinical outcome. This pathology is related to a long exposure to asbestos mainly during occupational activities. In a recent study describing the incidence, prevalence and survival of 17,688 rare thoracic tumors in Europe, MPM was the most common with 19 cases per million people per year, and presented the lowest survival after five years (5%) [1]. Lung cancer is, overall, the most frequent cancer type worldwide, in terms of both incidence and mortality. The most common histologic subtype is adenocarcinoma (ADCA), comprising at least half of all lung cancers [2]. Regarding treatment, first-line chemotherapy approved for MPM pathologies uses a cisplatin-pemetrexed combination [3], whilst platin-based adjuvant chemotherapy after surgery is employed in the treatment of lung ADCA [4]. However, the efficiency of these chemotherapies gave very little benefit regarding clinical outcome. Therefore, new treatments and/or new therapeutic strategies are highly needed, such as drugs combinations including platin derivatives.

It has recently been reported that these pathologies present specific epigenetic modifications [5-6] which would make them sensitive to ‘epigenetic’ modulators. Thus, several clinical trials using such drugs are being undertaken [7-8]. Epigenetic modifications are early and common events during the malignant transformation of cells. Amongst these, histone acetylation controls gene transcription by regulating chromatin compaction [9]. Deregulation of the histone acetyl transferases (HATs) and histone deacetylases (HDACs) balance has been observed in numerous cancer cells [10-11] resulting in histone hypoacetylation, repression of tumor suppressor genes (TSG) expression and functional alteration of proteins regulated by acetylation, such as p53 [12]. To counteract this phenomenon, HDAC inhibitors (HDACi) are used to re-induce histone acetylation and, thus, TSG expression, with success in a large number of cancer types [13]. These compounds were demonstrated to induce principally cell-cycle arrest and apoptosis or sensitization to apoptosis.

Numerous HDACi have been developed to improve the antitumor activities of this class of chemotherapeutic molecules. These compounds are classified into four families - hydroxamic acids, benzamides, depsipeptides, and short-chain fatty acids - depending on their chemical structures [14]. Two HDACi are approved by the FDA for the treatment of cutaneous T-cell lymphoma (CTCL) – SAHA (hydroxamic acid, vorinostat) [15] and romidepsin (depsipeptide) [16]. However, a large phase III clinical trial on mesothelioma using SAHA demonstrated no clear benefit on the overall survival compared with the placebo group (*unpublished*, http://goo.gl/dZCFx). This result adds further weight to the limitations already shown by clinical trials performed on solid tumors with HDACi and notably SAHA, relating to a short plasma half-life, poor diffusion in tumor tissues and hematological toxicity [17], leading to poor efficiency [8]. Thus, different strategies have been developed to compensate for these deficiencies and to take advantage of the antitumor properties of HDACi based mainly on the combination of these drugs with current chemotherapeutic agents.

Using a BRET (bioluminescence resonance energy transfer)-based screening assay, we previously identified new HDACi derived from Trichostatin A - two pan-HDACi (ODH and NODH) and two class I HDACi (ODB and NODB) - with potent histone H3 acetylation-inducing properties [18]. Our work demonstrated that NODH is active at nanomolar concentrations and shows an increased duration of histone H3 acetylation in comparison to SAHA and the newly identified HDACi ODH, ODB and NODB [18-19]. In the present work, we aimed to investigate the antitumor effects of our new HDACi compounds, compared with SAHA as a reference, alone or in combination with cisplatin. First, we analyzed the mRNA expression of HDAC in MPM and in lung ADCA cell lines in order to identify a particular modification of HDAC or class of HDAC expression which could lead to the selection of an appropriate family of HDACi. Then, the effects of our compounds were studied on cell-cycle, apoptosis and on the selection of ‘less-sensitive’ cells. We also compared these compounds for their effects on target-tumor-cell genes expression. Finally, we combined the HDACi with cisplatin to determine whether this combination can further decrease cell viability and growth rate of MPM and lung ADCA cells compared with the effects observed using the compounds individually. To evaluate the toxicity of this strategy on healthy cells, we tested the combination on primary mesothelial cells

## RESULTS

### Expression profiles of class I and class II HDAC in MPM and lung ADCA cell lines and in normal mesothelial cells

**Figure 1:**
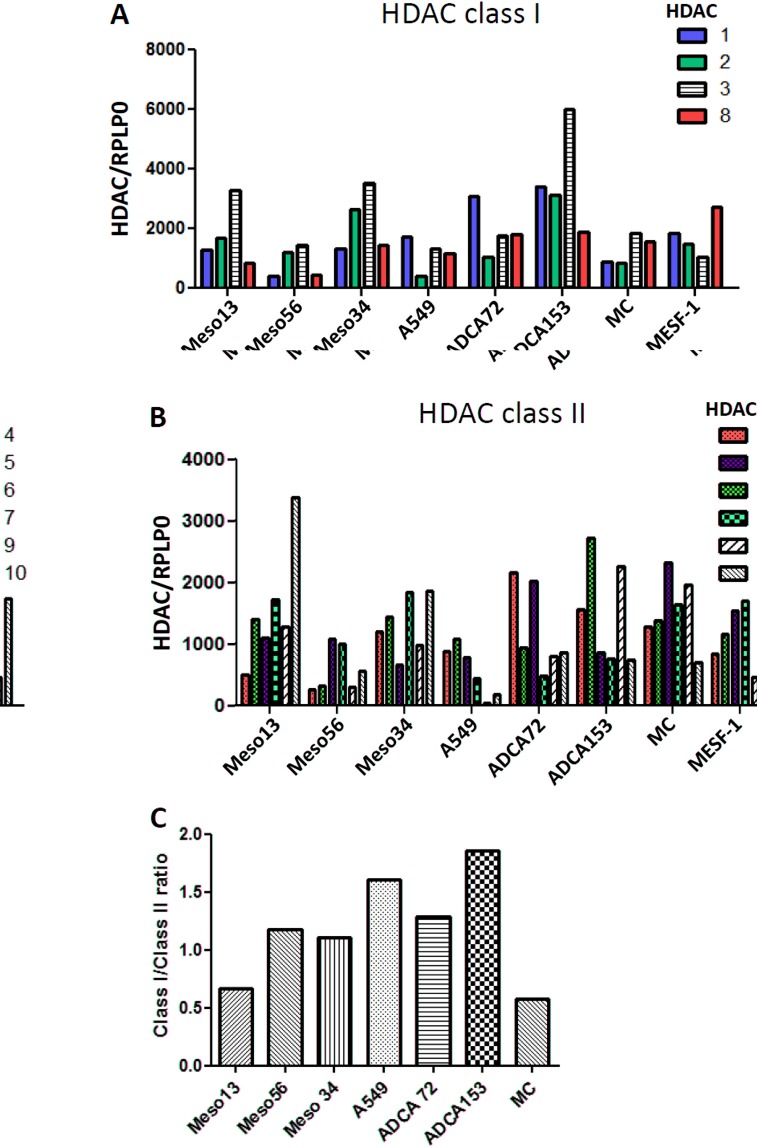
Expression of HDAC in MPM, lung ADCA and mesothelial cells (MC) Class I (A) and Class II (B) HDAC expression for MPM and lung ADCA cells were determined using real-time PCR. Individual mRNA levels were normalized to RPLP0 (ribosomal phosphoprotein P0 housekeeping gene). C), HDAC classI/classII expression in the cancer cells.

In order to identify a particular modification of HDAC or class of HDAC expression in our cell lines which could lead to the selection of an appropriate family of HDACi, we first determined by RT-PCR the expression profiles of HDACs in 3 MPM and 3 lung ADCA human cell lines. Normal pleural mesothelial cells (MC) and the commercial peritoneal mesothelial cells, MESF-1, were used as control cells. The expression of class I (Fig.1A) and class II (Fig.1B) HDACs were different and heterogeneous for each cell line tested. The same observation was made between MESF-1 and pleural mesothelial cells which could arise from their origin. However, we noted that the class I/class II HDAC expression ratio (Fig.1C), was higher for all cancer cell lines, except Meso13, when compared with normal pleural mesothelial cells (MC). This observation was already made for lung cancer cells but not for MPM cells. In order to confirm this tendency in MPM cells, we extended the analysis to 18 MPM cell lines and to 2 independent isolations of pleural mesothelial cells. Results showed, for the first time, a significant increase of the class I/class II HDAC expression ratio in MPM cells compared to primary pleural mesothelial cells (Fig.S1A). HDAC 3 seemed also to be highly expressed by MPM cell lines compared to normal mesothelial cells however, this observation was not confirmed on our MPM mRNA biocollection (Fig.S1B).

These results demonstrate that the MPM and lung ADCA cell lines tested here have different HDAC mRNA expression profiles. Thus, both class I-specific and pan-HDACi can be used on these tumor cells whereas compounds with highly restrictive HDAC specificity should be not appropriated.

### Sensitivity of tumor cell lines to new HDACi

To evaluate the sensitivity of tumor cells to these new HDACi compounds, we first defined the HDACi IC_50_ for cell-growth inhibition. We performed dose-response experiments and measured cell viability. The results are summarized in Table 1. All of the tested cell lines were more sensitive to NODH, active in the nanomolar range, than to ODB, NODB, ODH or SAHA active in the micromolar range. We cannot conclude on a particular selective effect of compounds between MPM and lung ADCA cell lines regarding the high variability in their activities between cells.

**Table 1:** IC_50_ values for HDACi-induced inhibition of cell growth

Cell lines	ODB μM	NOBD μM	ODH μM	NODH nM	SAHA μM
Meso 13	3.88 ± 0.29	1.07 ± 0.28	0.74 ± 0.11	17.84 ± 8.09	0.60 ± 0.17
Meso 56	0.68 ± 0.07	0.44 ± 0.10	0.18 ± 0.08	10.18 ± 3.05	0.44 ± 0.08
Meso 34	8.00 ± 0.28	4.35 ± 0.31	1.69 ± 0.17	36.46 ± 13.88	1.61 ± 0.03
ADCA 153	3.96 ± 0.28	1.31 ± 0.26	0.86 ± 0.11	6.32 ± 1.27	0.53 ± 0.08
ADCA 72	15.87 ± 0.26	3.79 ± 0.22	0.56 ± 0.06	10.67 ± 3.88	0.49 ± 0.10
A549	16.00 ± 0.15	3.99 ± 0.27	1.74 ± 0.14	31.93 ± 7.34	2.31 ± 0.26

IC_50_ values were determined using GraphPad prism, Prism 5 for Windows, by curve fitting using a sigmoidal dose response model. Results are the means ± S.E.M of three independent experiments.

### Apoptosis induction and cell-cycle alteration with hydroxamate and benzamide compounds

**Figure 2:**
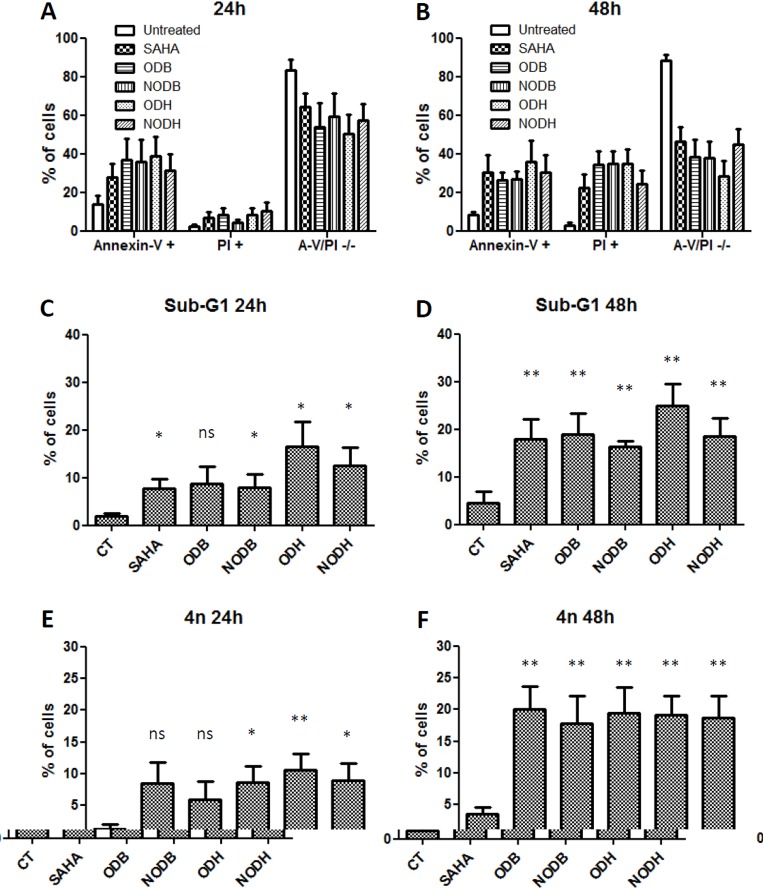
Measurement of apoptosis and cell-cycle analysis of the tumor cells treated with ODB, NODB, ODH and NODH MPM and lung ADCA cells were treated with SAHA, ODB, NODB, ODH or NODH (5 IC_50_) for 24 h or 48 h. Apoptosis measurements were performed after 24 h (A) or 48 h (B) of treatment using annexin-V-FITC and propidium iodide labeling followed by flow cytometry analysis. Cell cycles were studied after 24 h (C and E) or 48h (D and F) of treatment using propidium iodide labeling of cells fixed with 70% ice-cold ethanol followed by flow cytometry analysis. C and D, percent of cells in sub-G1 and, E and F, percent of cells with 4n of DNA. Results are expressed as the means +/− S.E.M. of the results obtained with the six cell lines studied. *; p < 0.05, **; p < 0.01 and ns; non significant.

In order to characterize the drug effects on tumor-cell-death resistance, we analyzed apoptosis and the cell cycle (Fig.2). After 24h treatment with 5×IC_50_ of each drug on the six cell lines, we observed 35% annexin-V-FITC–positive cells (apoptosis) and 10% necrotic cells (Propidium Iodide positive) (Fig.2A). Whereas the apoptotic cell proportion did not change after 48h treatment, the proportion of necrotic cells reached 35% (Fig.2B), which probably indicated secondary necrosis.

In addition, we performed cell-cycle analysis using PI labeling of permeabilized cells. After 24h treatment, the main observed perturbations were an increased proportion of cells in sub-G1 (Fig.2C) and a large proportion of cells exhibiting more than 4n of DNA (Fig.2E). These changes increased after 48h treatment and were significant for all the tested compounds (Fig.2D and 2F).

These results demonstrated that NODH at nanomolar concentrations and the others compounds at micromolar concentrations induced similar level of tumor cells apoptosis consecutively to cell cycle perturbations.

### Increased expression of HDACi target genes with new compounds

**Figure 3:**
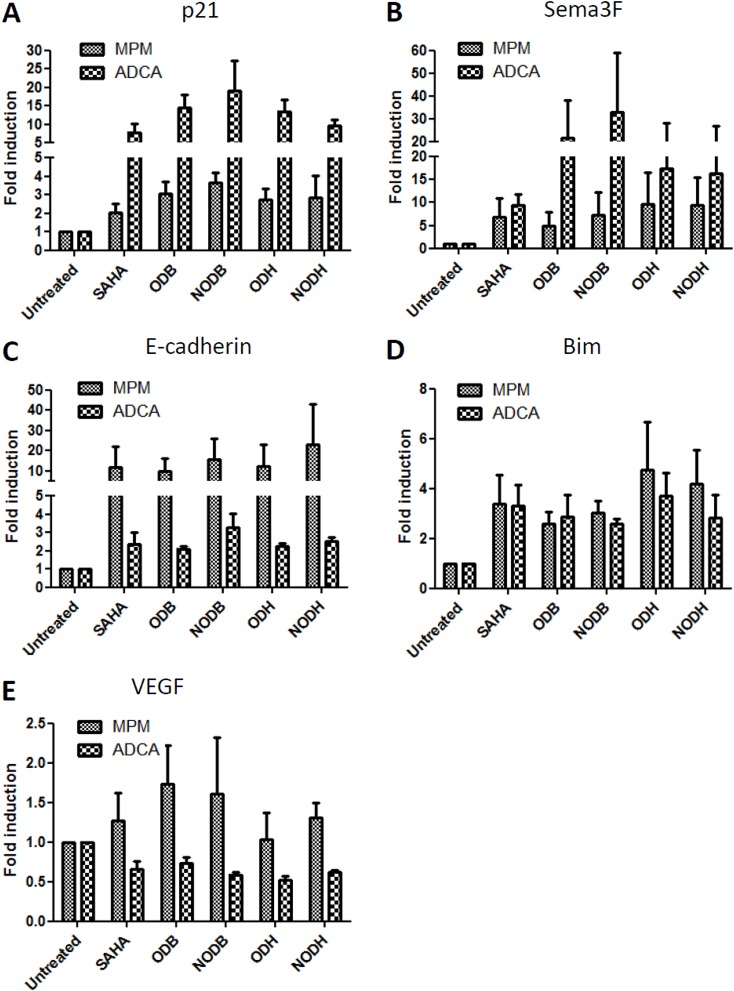
Measurement of target gene expression following HDACi treatments MPM and lung ADCA cells were treated with SAHA, ODB, NODB, ODH or NODH (5 IC_50_) for 24 h. mRNA expression of A) p21, B) Semaphorin 3F (Sema3F), C) E-cadherin, D) Bim and E) VEGF were measured using real-time PCR. Individual mRNA levels were normalized to RPLP0 (ribosomal phosphoprotein P0 housekeeping gene). Results are expressed as the means +/− S.E.M. of results obtained with the three MPM cell lines or with the three lung ADCA cell lines.

To elucidate some of the mechanisms involved in antitumoral effects of these HDACi, we measured the expression of known target genes: Semaphorin 3F for tumor suppression functions, p21 for cell cycle control, Bim for apoptosis and E-cadherin for epithelial-mesenchymal transition. Cells were treated with the different HDACi for 24h. The mRNA expression of p21 (Fig.3A), Semaphorin3F (Fig.3B), E-cadherin (Fig.3C) and Bim (Fig.3D) were increased following the different HDACi treatments. We also measured the expression of the vascular endothelial growth factor (VEGF), which acts as an autocrine growth factor for MPM [20] and lung ADCA cells [21]. VEGF gene expression was reduced in ADCA cell lines, whereas a tendency to upregulation was observed in MPM cells (Fig.3E).

### Combination of HDACi with cisplatin decreases MPM and ADCA cell viability and limits the emergence of ‘less-sensitive’ tumor cells

**Figure 4:**
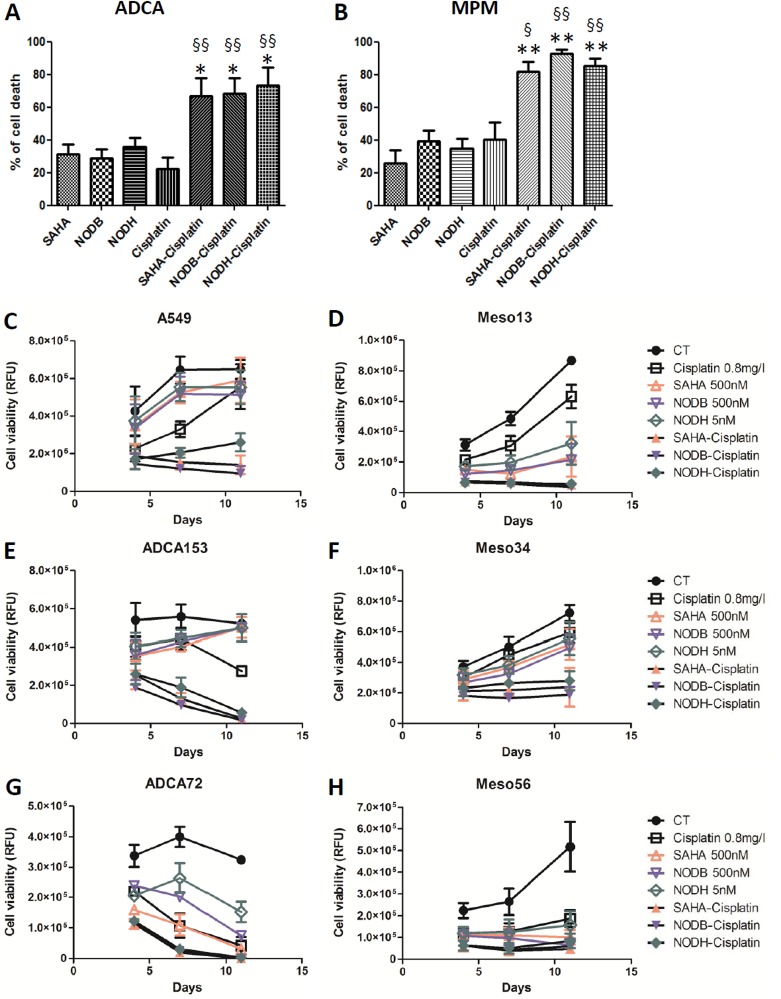
Evaluation of the HDACi-cisplatin combination on MPM and lung ADCA cell viability Lung ADCA (A, C, E and G) and MPM cells (B, D, F and H) were treated with SAHA (500nM), NODB (500nM), NODH (5nM) for 24 h prior addition of cisplatin (0.8 mg/l) for 72 h. For C to H, cycles of treatment were performed three times. After each cycle of treatment, cell viability was determined using Uptiblue reagent. Results are expressed as the means +/− S.E.M. of three independent experiments. HDACi vs HDACi-cisplatin, **: p<0.01. Cisplatin vs cisplatin-HDACi, §: p<0.05; §§: p<0.01

Previous studies showed that a combination of cisplatin with valproic acid (VPA), a class I and class IIa HDACi active at millimolar concentrations, improved the antitumor effect on MPM cells compared with the drugs used alone [22-23]. Here, we tested the combination of cisplatin with SAHA, with NODB or with NODH. Drug concentrations were chosen to induce approximately 30% cell death when used alone. Cells were pretreated with HDACi 24h prior to cisplatin addition. When combined, the drugs induced more than 70% of lung ADCA cell death (Fig.4A) and more than 80% of MPM cell death (Fig.4B). The efficiency of the combination on the emergence of ‘less sensitive’ cells was assessed by treating cells three times over a period of eleven days. All cells were treated with the same concentrations of SAHA (500 nM), NODB (500 nM), NODH (5 nM) and cisplatin (0.8 mg/l), alone or in combination (Fig.4C to H). An increase of the relative fluorescence unit (RFU), corresponding to an increase in cell number, was observed in the untreated condition for all lung ADCA cell lines, reaching a plateau from the sixth day (confluence) of culture. For the MPM cell lines treated with drugs alone, an increase of the RFU was observed over the eleven-day culture period. We observed the emergence of HDACi-less sensitive cells for lung ADCA as early as the sixth day of treatment for all tested cells except for ADCA72. When treated with cisplatin alone, the MPM cell lines and A549 only presented a ‘less sensitive’ phenotype as soon as the sixth day after treatment. Interestingly, the HDACi/cisplatin combination prevented the emergence of ‘less-sensitive’ cells and/or induced a better control of cell growth for all tested cell lines.

### Combination of HDACi with cisplatin presents a lower toxicity on normal mesothelial cells than on cancer cells

**Figure 5:**
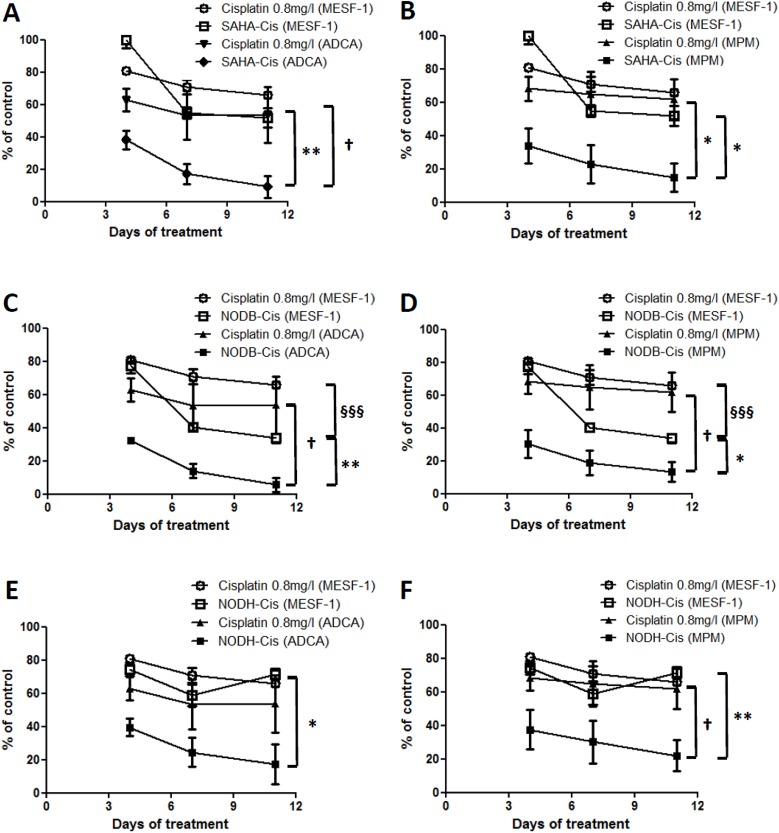
Evaluation of the HDACi-cisplatin combination on primary mesothelial cell viability MESF-1 were treated with SAHA (500nM) (A, B), NODB (500nM) (C, D), NODH (5nM) (E, F) for 24 h prior addition of cisplatin (0.8mg/l) for 72 h. Cycles of treatment were performed three times. After each cycle of treatment, cell viability was determined using Uptiblue reagent. Graphics represent results obtained with MESF-1 compared with those obtained with ADCA (A, C and E) or MPM cells (B, D and F). Results are expressed as the means +/− S.E.M. of three independent experiments. *: comparison between MESF-1 and tumor cells. *: p<0.05; **: p<0.01. §: Comparison between MESF-1 conditions. §§§: p<0.001. †: Comparison between tumor cells conditions. †: p<0.05

The potential of this combination strategy was evaluate by determining the toxicity of the combinations on normal mesothelial cells. The same doses used for tumor cells were used to treat MESF-1 cells. The treatments were repeated 3 times and cell viability was determined before each repetition of the treatments. The results showed that in all cases, the cisplatin toxicity is not different between MESF-1 and cancer cells (approximately 30 to 40% cell death at the end of the experiment) (Fig.5). Whereas the combination NODB-cisplatin (Fig.5C and 5D) is significantly more toxic than cisplatin alone on MESF-1 (cisplatin: 30% cell death and NODB-cisplatin: 60% cell death), the combinations SAHA-cisplatin (Fig.5A and 5B) and NODH-cisplatin (Fig.5E and 5F) presented no additional toxicity when compared to cisplatin alone. Interestingly, SAHA- and NODH-cisplatin combinations induced a strong toxicity on lung ADCA (approximately 85% cell death) and MPM cells (approximately 80% cell death) compared the one induced on MESF-1 cells (40% cell death for SAHA-cisplatin and 30% cell death for NODH-cisplatin).

## DISCUSSION

Several clinical trials have demonstrated the limitations of HDACi compounds, notably in the treatment of solid tumors. An improvement of the pharmacological properties of these drugs, or their use in combination with standard chemotherapeutic agents, could be of interest to increase their clinical benefit.

In this study we demonstrated for the first time that HDAC expression is modified in MPM cells compared with normal pleural mesothelial cells. We also characterized the antitumor effects of four newly synthesized HDACi in comparison with SAHA, one of the two HDACi approved by the FDA for the treatment of cutaneous T-cell lymphoma. These effects were analyzed alone, or in combination with cisplatin, on primary mesothelial cells, MPM and lung ADCA cell lines. We demonstrated that the same effects obtained for SAHA at micromolar concentrations can be obtained with our hydroxamate compound NODH (pan-HDACi) at nanomolar concentrations. By combining HDACi with cisplatin, we showed that this strategy facilitates the prevention or limitation of the emergence of ‘less sensitive’ phenotypes in MPM and ADCA cell lines whereas poor toxicity was observed on primary mesothelial cells.

The expression profile of HDAC was shown to be modified in many cancer cells and in tissues [10-11]. This observation can have a strong impact on the selection of the appropriate HDACi to use in lung or MPM cancer treatments. In the present work, we observed that all MPM and lung ADCA cell lines presented different HDAC expression profiles. The main change observed in MPM cells, as compared with normal pleural mesothelial cells, was a significant increase of the class I/class II HDAC ratio. This observation was also found in lung ADCA cells, confirming previous studies [24-25]. HDAC expression profile changes have been associated with a poor prognosis in some cancers, notably in lung cancer [24-25]. Therefore, the modifications of HDAC expression observed in MPM cells could also be associated with the tumor-aggressiveness and poor survival of MPM patients. These modifications are related to a global increase of class I HDAC expression for Meso13, Meso34, ADCA72 and ADCA153, or to a global decrease of classII HDAC expression for Meso56 and A549 (Fig.S2). These results suggest that pan-HDACi and class I HDACi would be more appropriate to treat MPM and lung ADCA cells than would highly specific HDAC inhibitors.

Independently of HDAC expression profile, all the tested compounds induced the formation of cells with an increased DNA content associated with an increase of apoptotic cells. This observation suggests that treatments interfere with the mitotic process probably by leading to a failure of chromosome congression and cytokinesis, as previously described [30-33], and then to apoptosis of cells. The functional effects of the compounds were correlated with the induction of expression of the usual HDACi target TSG p21, E-cadherin, Bim and Sema3F, implicated in control of the cell cycle, apoptosis and tumor progression [34-36]. Using antibody array, we confirmed induction of the expression of p21 and Bim at the protein level by NODH, NODB and SAHA and, moreover, we noted an induction of the expression of proteins implicated in intrinsic and extrinsic pro-apoptotic pathways, such as TRAIL-R, HTRA, Bim, SMAC, caspase-8 and caspase-3 (Fig.S3). These results need to be confirmed by a large biochemical study to accurately quantify the protein expressions and to decipher with the pathways differentially regulated by the compounds. These observations correlate with previous works performed on MPM and lung cancer models in mice, which demonstrated a sensitization of tumor cells to chemotherapeutic agents using HDACi. This suggests that our compounds could sensitize cancer cells to apoptosis-inducing treatments. We also evaluated the anti-angiogenic properties of the drugs by measuring the mRNA expression of VEGF, which has been described as a downregulated target gene by HDACi in lung cancer cells [37]. Whereas VEGF expression was strongly reduced in all ADCA cell lines with all the compounds, a high variability was observed in MPM cell lines. This suggests that the use of HDACi to target VEGF expression in MPM should be ineffective compared with lung ADCA.

Current chemotherapies to treat MPM and lung ADCA are mainly based on the use of platin derivatives [3, 38]. The clinical benefits of these therapies, in combination with an antifolate, are still limited, particularly in MPM [39-40]. In addition, clinical studies of HDACi treatment alone on solid tumors have demonstrated poor improvement of the clinical outcome mainly due to a poor stability of HDACi in plasma and hence poor diffusion into tumor tissues [8]. Thus, new strategies have been developed in which HDACi are used as sensitizers to other chemotherapeutic agents [13]. In the present investigation, we observed that our tested HDACi compounds in combination with cisplatin allowed a better control of the tumor cells growth than drugs alone. Regarding the induction of pro-apoptotic proteins expression by SAHA, NODB and NODH, we pretreated tumor cells with HDACi prior to the addition of cisplatin. Interestingly, the combination of HDACi with cisplatin, using doses that induce approximately 30% cell growth reduction when added alone for 72h, resulted in a cell-growth reduction ranging from 70% to 90%. An improved effect of cisplatin when combined with HDACi was observed on all tested cell lines. Indeed, combination completely eliminated ADCA153 and ADCA72 cells after eleven days of treatment and drastically decreased the growth of A549 and MPM cells compared with use of the molecules alone. These results correlate with previous studies performed on mouse models of human MPM and lung cancer using valproic acid [22-23]. SAHA was already described to sensitize mesothelioma cells to a single cisplatin treatment [41]. However, here, we show for the first time that the benefit of the combination SAHA-cisplatin, and also of our HDACi with cisplatin, was maintained over time, which avoid the generation of ‘less-sensitive’ cells observed when drugs were used alone. Indeed, the majority of tested cell lines, mainly MPM cells, became ‘less-sensitive’ to cisplatin which could explain its poor benefit on the clinical outcome of patients [39]. The efficacy of HDACi alone was also strongly limited due to the appearance of a systematic ‘reduced sensitivity’ phenotype after the second repetition of the treatment, except for ADCA72. This observation was supported by studies on cancer cells that show the existence of several mechanisms implicated in the ‘reduced sensitivity’ to HDACi which could be investigated in MPM and lung ADCA cells [26-29].These mechanisms associated with the poor stability in plasma and the poor diffusion in tumor tissues of HDACi could probably be responsible for their disappointed effect in clinic. Moreover, here we show that the toxicity of the SAHA-cisplatin and NODH-cisplatin combinations is significantly lower on primary Human mesothelial cells than on cancer cells demonstrating the selectivity of these combinations towards cancer cells. This selectivity towards cancer cells could be driven by HDACi regarding their absence of toxicity on MESF-1 whereas an induction of 40% to 50% cell death was observed on cancer cells (Figure S4).

The strategy combining cisplatin and SAHA, with or without additional drugs on solid tumor is under evaluation in phase I clinical trials (http://clinicaltrials.gov/ct2/home). Our work supports this strategy regarding the better control of MPM and lung ADCA cells growth obtained with this combination compared with the compounds alone. Our results obtained from in vitro experiments on human cells also suggest that an administration in the pleural cavity, in the case of MPM, could be considered regarding the poor toxicity of the SAHA and NODH-cisplatin combination on primary Human mesothelial cells. The improved pharmacological properties of our new HDACi, NODH, need to be confirmed *in vivo* in order to evaluate the real potential of this epigenetic modulator.

Altogether, our results demonstrate the antitumor potential of NODB and mainly NODH compounds, which present interesting pharmacological properties and antitumor properties compared with SAHA. Moreover, our work supports the proposition that cisplatin in combination with HDACi could be of real interest in the treatment of these pathologies and that NODH could be an alternative to existing HDACi regarding its improved pharmacological properties.

## METHODS

### Drugs

SAHA (suberoylanilide hydroxamic acid) was purchased from R&D chemicals. ODH (4-methyl-5-(2-methyl-3oxo-2,3-dihydro-benzofuran-2-yl)-penta-2,4-dienoic acid hydroxamide), ODB (4-methyl-5-(2-methyl-3oxo-2,3-dihydro-benzofuran-2-yl)-penta-2,4-dienoic acid benzamide), NODB (5-(6-dimethylamino-2-methyl-3oxo-2,3-dihydro-benzofuran-2-yl)-4-methyl-penta-2,4-dienoic acid benzamide) and NODH (5-(6-dimethylamino-2-methyl-3oxo-2,3-dihydro-benzofuran-2-yl)-4-methyl-penta-2,4-dienoic acid hydroxamide) were prepared as described previously [19].

### Cell culture

The human lung cancer cell line, A549, was obtained from the American Type Culture Collection (ATCC). The mesothelioma, Meso34, Meso56 and Meso13, and lung adenocarcinoma (ADCA), ADCA153 and ADCA72, cell lines were established from pleural fluids of patients [42]. Isolation and culture of normal mesothelial cells were described previously [42]. All cell lines were maintained in RPMI medium (Invitrogen) supplemented with 2 mM L-glutamine, 100 IU/ml penicillin, 0.1 mg/ml Streptomycin and 10% heat-inactivated fetal calf serum (FCS) (Eurobio) and cultured at 37°C in a 5% CO_2_ atmosphere. The primary peritoneal mesothelial cells, MES-F, were purchased from Tebu-bio biosciences and cultured according to the manufacturer's recommendations.

### RNA isolation and real-time RT-PCR

Total RNA was isolated using the Nucleospin^®^ RNAII Kit according to the manufacturer's protocol (Macherey-Nagel). One microgram of total RNA was reverse-transcribed using Moloney murine leukemia virus reverse transcriptase (Invitrogen). Real-time PCR (RT-PCR) was carried out using an Mx3500P thermocycler (Stratagene). PCR reactions were performed using QuantiTect Primer Assays (Qiagen) and the RT² Real-Time SYBR-Green/ROX PCR Mastermix (Qiagen), according to the manufacturer's instructions. The relative amount of the target RNA, called the starting quantity (SQ), was determined using the Mx4000 software, by comparison with the corresponding standard curve for each sample performed in duplicate. Each transcript level was normalized by division with the expression values of the acidic ribosomal phosphoprotein P0 housekeeping gene (*RPLP0*), used as an internal standard.

### Determination of cell viability

Cell viability was monitored using Uptiblue (Interchim). Cells were seeded in 96-well plates at a density of 5×10^3^ cells/well in culture medium. Twenty-four hours later, compounds were added for 72 h. Uptiblue reagent (5%, v/v) was then added to the culture medium for 2 h at 37°C. Fluorescence was measured at 595 nM after excitation at 532 nM using a Typhoon apparatus (GE Healthcare). For kinetic experiments, culture medium containing Uptiblue was replaced with medium containing the drugs or without drug as control for 72 h and the procedure for cell viability measurement was repeated twice. Results were expressed as the percentage of the untreated cells.

### Detection of apoptosis

Cells were seeded at a density of 1×10^6^ cells/well in 6-well plates and treated with doses corresponding to five times the IC_50_ of SAHA, ODB, NODB, ODH or NODH, as determined in cell viability experiments (Table 1). After 24h or 48h culture, floating and adherent cells were combined, labeled using the Annexin V-fluorescein-isothiocyanate (FITC) apoptosis detection kit (*Becton Dickinson*) following the manufacturer's instructions, and analyzed by flow cytometry (FACSCalibur; Becton Dickinson). Ten thousand events were collected and analyzed with the FACS Flowjo Software (Tree Star Inc).

### Cell cycle analysis

Cells were seeded at a density of 1×10^6^ cells/well in 6-well plates and treated with doses corresponding to five times the IC_50_ of SAHA, ODB, NODB, ODH or NODH as determined in cell viability experiments (Table 1). After 24 h or 48 h culture, cells were trypsinized, collected by centrifugation at 500 *g* for 10 min, washed once with PBS and fixed with cold 70% ethanol. After incubation at −20°C for at least 1 h, cells were washed once with PBS, resuspended in PBS containing RNAse A (200 μg/ml; Invitrogen) and propidium iodide (2.5 μg/ml; Sigma Aldrich) and analyzed by flow cytometry (FACSCalibur; Becton Dickinson). Cell doublets were excluded from the analysis using the (FSC-H/FSC-W) gating method. Ten thousand events were collected and analyzed with the FACS Flowjo Software.

### Expression of proteins implicated in apoptosis following HDACi treatment

Cells were seeded at a density of 1×10^6^ cells/well in 6-well plates and treated with doses corresponding to five times the IC_50_ of SAHA, NODB or NODH as determined in cell viability experiments (Table I). After 24 h, cells were lysed in 300 μl Raybiotech lysis buffer containing freshly added protease inhibitors (Complete, Roche). Samples were sonicated for 15 min at 60 KHz wavelength using a Bioruptor^®^ (Diagenode). After centrifugation at 8,000 g for 5 min at 4°C, protein concentrations were determined using a protein quantitation kit from Interchim. One hundred micrograms of each MPM lysate or lung ADCA lysate were pooled. Protein expression analysis was performed using the Raybio® Human Apoptosis Antibody Array Kit (Raybiotech) according to manufacturer's instructions.

### Statistical analysis

Statistical analyses were performed using GraphPad prism, Prism 5 for Windows. Data are expressed as the means ± S.E.M. of at least three experiments. Statistical comparisons were made using an unpaired t test.

## SUPPLEMENTARY FIGURES


