# Supplementary material for: New histone deacetylase inhibitors improve cisplatin antitumor properties against thoracic cancer cells
Source: Oncotarget. 2014 Jun 3;5(12):4504–15. doi: 10.18632/oncotarget.2056 (PMC4147341; doi:10.18632/oncotarget.2056)
Supplement: Supplementary file 1 [file oncotarget-05-4504-s001.pdf]

New histone deacetylase inhibitors improve cisplatin antitumor properties against thoracic cancer cells

Supplementary Material

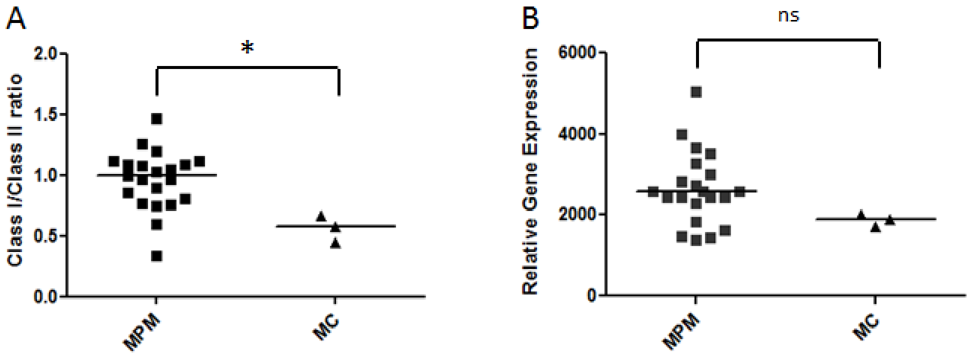

Figure S1: HDAC mRNA expressions in MPM and in primary mesothelial cells (MC).
ClassI and classII HDAC expression ratio (A) and HDAC 3 expression (B) in 21 MPM and 3 MC cells were determined using real-time PCR. Individual mRNA levels were normalized to RPLP0 (ribosomal phosphoprotein P0 housekeeping gene). *; $p < 0.05$, ns; non significant.

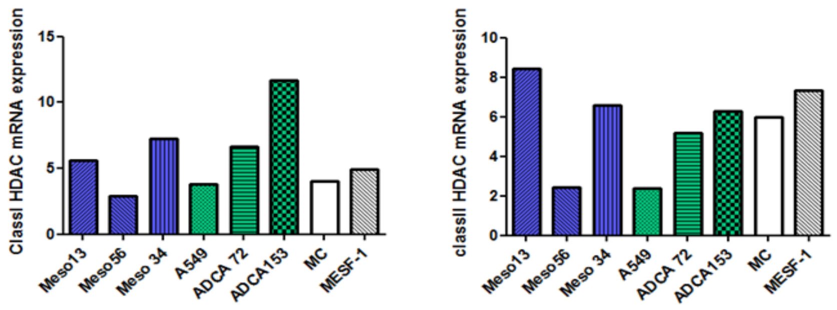

Figure S2: Expression of classI and classII HDAC in MPM and lung ADCA, and in normal mesothelial cells (MC).
Class I (left) and Class II (right) HDAC expressions in Meso 13, Meso 34, Meso 56, A549, ADCA 72, ADCA 153 and MC cells were determined using real-time PCR. Individual mRNA levels were normalized to RPLP0 (ribosomal phosphoprotein P0 housekeeping gene). mRNA expressions of class I and class II were represented as the addition of each HDAC expression of one class.

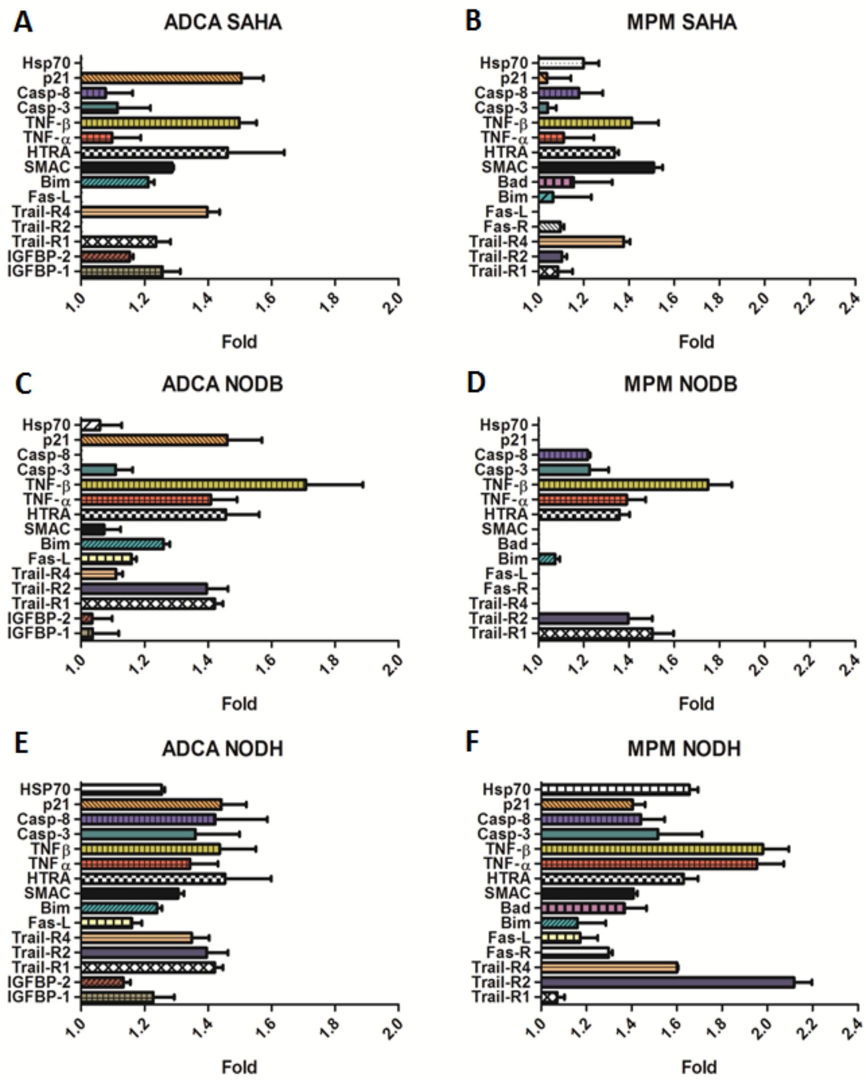

Figure S3: Measurement of the expression of proteins implicated in apoptosis pathways following HDACi treatments.

Lung ADCA (A, C and E) and MM cell lines (B, D and F) were treated with SAHA (A and B), NODB (C and D), or NODH (E and F) ($5EC_{50}$) for 24 h. Induction of the expression of proteins implicated in apoptosis pathways was studied using the Raybio® Human Apoptosis Antibody Array Kit. Graphics represent the proteins significantly regulated following HDACi treatments.

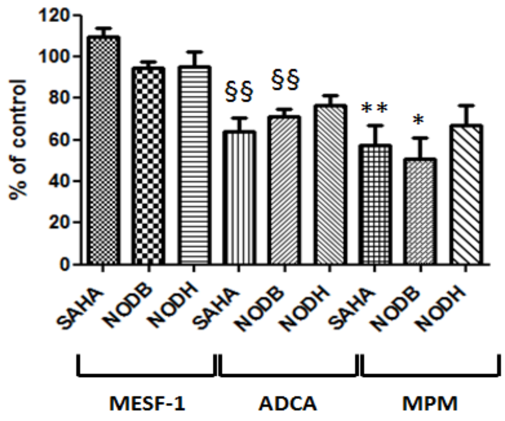

Figure S4: Evaluation of MESF-1 sensitivity to HDACi compared to ADCA and MPM cells.
MESF-1, lung ADCA and MPM cell were treated with SAHA (500 nM), NODB (500 nM), or NODH (5 nM) for 72 h. Cell viability was measured using Uptiblue cell counting reagent. Results are the means ± S.E.M. of three independent experiments. §: comparison between MESF-1 and lung ADCA cells. §§: p<0.01. *: comparison between MESF-1 and MPM cells. *: p<0.05; **: p<0.01.
